# Radiotherapy dose–response analysis for diffuse large B-cell lymphoma with a complete response to chemotherapy

**DOI:** 10.1186/1748-717X-7-100

**Published:** 2012-06-21

**Authors:** Jennifer A Dorth, Leonard R Prosnitz, Gloria Broadwater, Anne W Beaven, Chris R Kelsey

**Affiliations:** 1 Department of Radiation Oncology, University Hospitals Seidman Cancer Center and Case Western Reserve University, 11100 Euclid Avenue, Cleveland, OH 44106, USA; 2 Department of Radiation Oncology, Duke Cancer Institute, Durham, NC, USA; 3 Cancer Statistical Center, Duke Cancer Institute, Durham, NC, USA; 4 Department of Medicine, Division of Medical Oncology, Duke Cancer Institute, Durham, NC, USA

## Abstract

**Objective:**

To examine the efficacy of different radiation doses after achievement of a complete response to chemotherapy in diffuse large B-cell lymphoma (DLBCL).

**Methods:**

Patients with stage I-IV DLBCL treated from 1995–2009 at Duke Cancer Institute who achieved a complete response to chemotherapy were reviewed. In-field control, event-free survival, and overall survival were calculated using the Kaplan-Meier method. Dose response was evaluated by grouping treated sites by delivered radiation dose.

**Results:**

105 patients were treated with RT to 214 disease sites. Chemotherapy (median 6 cycles) was R-CHOP (65%), CHOP (26%), R-CNOP (2%), or other (7%). Post-chemotherapy imaging was PET/CT (88%), gallium with CT (1%), or CT only (11%). The median RT dose was 30 Gy (range, 12–40 Gy). The median radiation dose was higher for patients with stage I-II disease compared with patients with stage III-IV disease (30 versus 24.5 Gy, p < 0.001). Five-year in-field control, event-free survival, and overall survival for all patients was 94% (95% CI: 89-99%), 84% (95% CI: 77-92%), and 91% (95% CI: 85-97%), respectively. Six patients developed an in-field recurrence at 10 sites, without a clear dose response. In-field failure was higher at sites ≥ 10 cm (14% versus 4%, p = 0.06).

**Conclusion:**

In-field control was excellent with a combined modality approach when a complete response was achieved after chemotherapy without a clear radiation dose response.

## Introduction

Diffuse large B-cell lymphoma (DLBCL) is the most common subtype of non-Hodgkin lymphoma. The initial treatment is combination chemotherapy, most commonly consisting of the anti-CD20 antibody rituximab combined with cyclophosphamide, doxorubicin, vincristine, and prednisone (R-CHOP). Rituximab improves both event-free and overall survival in patients with DLBCL.

After chemotherapy alone, the most common site of disease recurrence is at sites of initial disease involvement [1]. Most randomized trials [1-3], but not all, have shown that consolidation radiation therapy (RT) decreases the risk of disease recurrence in stage I-II DLBCL after CHOP chemotherapy. Both randomized [4,5] and retrospective [6,7] studies have also suggested a benefit in select patients with advanced disease. These studies predate the widespread use of rituximab as well as positron emission tomography (PET), which is often performed after chemotherapy to assess response. However, a large, retrospective analysis from MD Anderson suggested that consolidation RT also improves outcomes in patients of all stages who achieve a complete response by PET with R-CHOP [8]. Additionally, we reported a retrospective analysis demonstrating improved outcomes in patients with stage III-IV DLBCL (65% received R-CHOP) treated with consolidation RT after achieving negative post-chemotherapy imaging (PET/CT in 73%) [9].

The optimal RT dose for patients who achieve a complete response to R-CHOP chemotherapy based on PET imaging is unknown. The randomized trials described above employed RT doses ranging from 30–55 Gy and were conducted prior to the use of rituximab and PET imaging [1-5]. Only one randomized trial, also predating rituximab and PET imaging, directly compared different RT doses in DLBCL and found no difference between 30 versus 40–45 Gy relating to in-field control or event-free survival [10].

At Duke Cancer Institute (DCI), our general approach for patients with stage I-II DLBCL is to treat with consolidation RT to all sites of initial disease involvement with a minimum of 30 Gy, since that dose has curative potential even if chemotherapy is ineffective [11]. For stage III-IV disease, select patients receive consolidation RT to either all sites of original involvement or to select sites (e.g. large tumor masses), if treatment of all sites is impractical. Lower doses of consolidation RT are typically used for advanced stage DLBCL (20–30 Gy) at DCI. The rationale is that patients with advanced DLBCL typically receive more cycles of chemotherapy than those with early-stage disease, allowing for a lower dose of consolidation radiation therapy. Further, with more widespread disease necessitating larger treatment volumes, the dose of RT is lowered to minimize toxicity. Finally, if chemotherapy hasn’t been sufficiently effective, higher doses of radiation are unlikely to make up the difference, which differs from early-stage disease where RT alone is a potentially curative modality.

It is quite possible that consolidation RT doses less than 30 Gy confer equivalent in-field control in patients who achieve a complete response to chemotherapy with rituximab. In order to investigate this hypothesis further, herein we report a retrospective dose–response analysis.

## Methods

This DCI Institutional Review Board-approved study reviewed all patients who were treated for stage I-IV DLBCL between 1995 and 2009 at DCI who achieved a complete response to combination chemotherapy and were treated with consolidation radiation. These years were selected as the time period during which post-chemotherapy radiologic assessment became routine. Patients with refractory disease or who did not achieve a complete response were excluded. Additionally, patients with central nervous system involvement were also excluded.

The diagnosis of DLBCL was confirmed by hematopathologists at DCI according to the WHO classification [12]. Initial work up generally included computed tomography (CT), gallium and/or positron emission tomography (PET), bone marrow biopsy and lactate dehydrogenase (LDH) and other routine blood studies. Staging was based on the Ann Arbor classification [13]. The International Prognostic Index (IPI) was calculated for all patients (age, performance status, extranodal involvement, stage, and LDH) [14]. Patients received a variety of chemotherapy regimens including CHOP, CNOP (cyclophosphamide, mitoxantrone, vincristine, and prednisone) or other, with or without rituximab. All patients had imaging performed to assess response to chemotherapy (during and/or after completion of chemotherapy).

Gallium scans were performed during the early years of the study period. Planar whole body and single photon emission computed tomography scans of the chest and abdomen were obtained 72 or 96 hours after the intravenous administration of 8 mCi of Ga-67 citrate. PET imaging subsequently supplanted gallium scans. From 1996–2003, PET scans were performed on a GE Advance scanner (General Electric Medical Systems, Milwaukee, WI) and the PET images were reviewed with a concurrent CT. From 2003–2009, a Discovery ST PET/CT scanner (General Electric Healthcare) was used and the PET images were reviewed with the non-contrast-enhanced CT.

Post-chemotherapy functional imaging studies were interpreted by attending nuclear medicine radiologists and were scored as positive or negative based on visual analysis alone, in agreement with the consensus recommendations of the International Harmonization Project in Lymphoma [15]. Post-chemotherapy CT scans were interpreted as negative if there were no sites of residual lymphadenopathy greater than 1 cm. Patients achieving a negative interim PET/gallium scan did not routinely have functional imaging performed at the completion of chemotherapy. All patients who had a positive interim PET/gallium scan had an additional study performed at least 2 weeks after the last cycle of chemotherapy.

Patients received consolidation RT at the discretion of the treating medical and radiation oncologists. All patients were treated with photon energies of 4–15 MV. Consolidation RT was given 3–4 weeks after finishing chemotherapy to originally involved sites plus an appropriate margin, without specifically targeting clinically uninvolved sites [16].

### Statistical analysis

Actuarial in-field control, event-free survival and overall survival were calculated using the Kaplan-Meier product limit method [17]. Five-year survival estimates with 95% confidence intervals (CI) were calculated. In-field local control was defined as the absence of disease recurrence within the previously-administered RT field, timed from the date of completion of radiotherapy. Patients without an in-field progression were censored at the time of distant failure, death, or last follow-up date. Event-free survival was defined as the time from completion of chemotherapy until lymphoma progression or death as a result of any cause, whichever occurred first. Alive patients without progression were censored at date of last follow-up. Overall survival was defined as the time from completion of chemotherapy until death as a result of any cause. Patients who were still alive were censored at the date of last follow-up. Categorical patient characteristics were compared using the chi-square test; continuous patient characteristics were compared using the Wilcoxon Rank-Sum test.

Given that most patients had multiple sites of disease involvement at diagnosis, and sites sometimes received different prescribed radiation doses, an analysis was conducted by disease site. Sites were delineated based on definitions of the standard involved field [16]. Radiation dose bins were in 5 Gy increments, ranging from ≤20 Gy to 36–40 Gy.

## Results

Between 1995 and 2009, 174 patients were identified who were treated with radiation for stage I-IV DLBCL at DCI. The following patients were excluded from the present analysis: 5 treated for relapsed or refractory disease, 25 with a partial response to chemotherapy by PET/gallium, 10 without post-chemotherapy imaging, and 29 with central nervous system involvement. Therefore, 105 patients treated to 214 sites were identified who met the inclusion criteria.

Patient characteristics can be found in Table1. Most patients had stage I-II disease (69%). Chemotherapy primarily consisted of R-CHOP (65%), but other regimens were also utilized. The median number of chemotherapy cycles was 6 (interquartile range, 4–6). Post-chemotherapy imaging consisted of PET/CT in 88%, gallium and CT in 1%, and CT only in 11% of patients.

**Table 1:** Patient characteristics

**Characteristic, *n***	**All patients**
	**(*n* = 105)**
Age (years)	
Median	62
Range	19–85
Stage	
I-II	73
III-IV	33
IPI score, median	1
Size*	
<10 cm	84
≥10 cm	21
Site category	
Nodal	51
Extranodal	19
Both	35
Chemotherapy	
R-CHOP	68
CHOP	27
Other	10
Post-chemotherapy imaging	
PET/CT	88
Gallium	1
CT alone	11
RT dose (Gy)	
Median	30
Range	12-40

*Largest mass.

Radiation therapy was administered in 1.8 – 2 Gy fractions to a median dose of 30 Gy, ranging from 12 – 40 Gy. The median radiation dose was higher for patients with stage I-II disease compared with patients with stage III-IV disease (30 versus 24.5 Gy, p < 0.001).

### Overall outcomes

**Figure 1:**
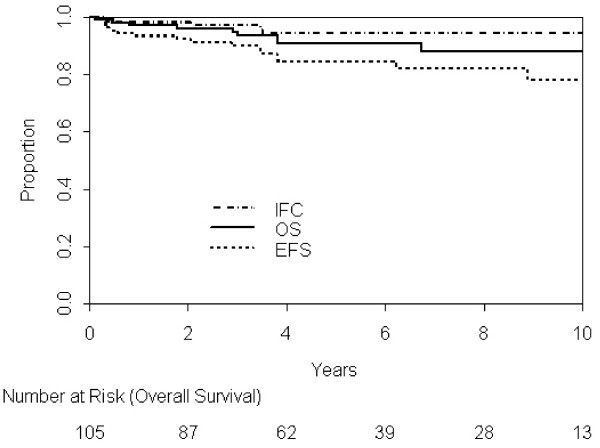
In-Field Control, Event-Free Survival and Overall Survival.

After a median follow-up for the alive patients of 5 years (IQR, 3–8 years), 13 patients relapsed. Five-year in-field control, event-free survival, and overall survival for all patients was 94% (95% CI: 89-99%), 84% (95% CI: 77-92%), and 91% (95% CI: 85-97%), respectively (Figure1). Among patients who relapsed, 2 relapsed only at initially-involved sites (stage I-II: n = 1; stage III-IV: n = 1), 8 at uninvolved sites (stage I-II: n = 5; stage III-IV: n = 3), and 3 in both involved and uninvolved sites (stage I-II: n = 2; stage III-IV: n = 1).

### Dose response analysis

Disease sites receiving lower doses of RT generally belonged to patients with higher IPI scores, reflecting the fact that lower RT doses were typically used for patients with advanced disease. The median IPI score was 2 among sites receiving 20 Gy, compared to 1 among sites receiving 30 Gy.

Prescribed radiation doses to individual sites and in-field failures can be found in Table2. Crude rates of local failure ranged from 0% to 6% without a clear dose response. Local recurrence appeared higher for sites ≥10 cm (14% versus 4%, p = 0.06). Patients with larger tumors who experienced in-field failure were treated with doses ≤ 30 Gy, but the number of events in each dose bin was small (Table2).

**Table 2:** In-Field Failures by Dose*

**Dose group**	**≤ 20 Gy**	**21-25 Gy**	**26-30 Gy**	**31-35 Gy**	**36-40 Gy**
Total sites, *n*	77	17	81	35	4
Sites failed, *n* (%)	5 (6%)	0 (0%)	4 (5%)	1 (3%)	0 (0%)
Subgroups					
< 10 cm					
Total sites, *n*	67	15	74	31	3
Sites failed, *n* (%)	3 (4%)	0 (0%)	2 (3%)	1 (3%)	0 (0%)
≥ 10 cm					
Total sites, *n*	10	2	7	4	1
Sites failed, *n* (%)	2 (20%)	0 (0%)	2 (29%)	0 (0%)	0 (0%)

*Crude rates of failure.

## Discussion

Randomized and retrospective studies have shown that consolidation RT significantly decreases the risk of disease recurrence in DLBCL, though most were not powered to detect an improvement in overall survival [1-8]. Given that many patients with DLBCL are long-term survivors, optimizing disease control while minimizing acute and late side effects is critical. The risk of RT-induced late effects, including development of secondary malignancies and cardiovascular disease, is undoubtedly related to the total dose utilized and volume treated [18].

The optimal RT dose for patients who have a complete response to R-CHOP chemotherapy based on PET imaging remains unknown. A randomized trial from the United Kingdom found excellent in-field control rates with RT doses as low as 30 Gy [10]. It is possible that RT doses less than 30 Gy are equally effective. Thus, we evaluated our own experience in patients with DLBCL treated predominantly with R-CHOP and all having negative post-chemotherapy imaging (88% assessed with PET). In this patient population, we observed excellent in-field control with lower doses of RT. Additionally, a relationship between RT dose and in-field control was not evident.

In the study from the UK, 640 patients with intermediate-to-high grade NHL (DLBCL: 85%; stage I-II: 86%) were randomized to 30 Gy versus 40–45 Gy [10]. Eighty percent of patients received consolidation RT as part of a primary combined-modality regimen, though 13% had relapsed or refractory disease and 10% were treated with palliative intent. At a median follow-up of 5 years, in-field control was similar between the low and high dose groups (82% vs 83%; HR 1.09, p = 0.7). Overall, in-field control was lower than that seen in retrospective series where 5 year rates were greater than 90% [19,20]. The inferior in-field control in the UK trial may be explained by inclusion of patients with refractory disease, 20% of patients receiving RT alone, and less rigorous radiographic assessment of chemotherapy response. It has been demonstrated that a positive post-chemotherapy PET scan compared to a negative scan predicts for worse 5-year in-field in patients who receive consolidation RT (71% vs 95%, p = 0.008) [20].

A retrospective series from the Netherlands suggests that RT doses less than 30 Gy may be equally effective in patients who have a complete response to chemotherapy [19]. This study found excellent in-field control rates among 94 patients with stage I intermediate-to-high grade NHL who achieved a complete response on CT imaging after 4 cycles of CHOP. There was no difference in 5-year crude in-field control between patients who received 26 Gy versus 40 Gy (92% vs 95%).

Similar to the study from the Netherlands, we found excellent 5-year in-field control of 94% among patients who had a complete response to chemotherapy and underwent consolidation RT. There was no difference in IFC among sites that received ≤ 20 Gy versus those that received 26–30 Gy (94% vs 95%). Our study is unique in that the majority of patients received R-CHOP (63%) and underwent functional imaging (89%). In addition, lower doses of RT were utilized (median, 30 Gy; range, 12–40 Gy).

In contrast to our findings, there was evidence of a RT dose response in a retrospective series from MD Anderson of 162 patients with stage I-III DLBCL [21]. Five-year in-field control was significantly worse for patients who received < 40 Gy compared with ≥ 40 Gy (83% vs 97%, p = 0.002). The main limitation of this study is that chemotherapy response assessment was based on plain X-ray imaging. Consistent with our study, there was a higher rate of in-field failure with tumors that were originally ≥ 10 cm.

A limitation of our study is that it is retrospective and non-randomized in nature. Further, there were few in-field failures, which may limit the power to detect a RT dose response. There was clearly a preference to treat more advanced stage patients with lower doses of RT. This may over- or underestimate a RT dose response because stage III-IV patients are at an overall higher risk of disease recurrence, but distant failures may eclipse the importance of in-field failures. We did not find the latter to be the case in that patterns of failure were similar between early and advanced stage patients. Further, the role of consolidation RT in advanced DLBCL is controversial and the benefit of radiation therapy, regardless of dose, has not been conclusively demonstrated. At DCI, we are currently treating DLBCL patients who have a complete response to 4 or more cycles of R-CHOP based on PET imaging with consolidation RT dose of 18–20 Gy as part of a phase II trial.

## Conclusion

In-field control was excellent in DLBCL patients who have a complete response to chemotherapy and receive consolidation RT. There were low rates of in-field failure after consolidation RT, irrespective of dose.

## Competing interest

The authors have declared no conflicts of interest.

## Authors’ contributions

JD and CK coordinated the study. JD: data collection. GB: statistical analysis. JD, LP, GB, AB, CK: manuscript preparation. All authors read and approved the final manuscript.
